# Assessment of factors associated with potential drug-drug interactions in patients with tuberculosis and HIV/AIDS

**DOI:** 10.1590/0037-8682-0103-2021

**Published:** 2021-07-23

**Authors:** Natália Helena de Resende, Silvana Spíndola de Miranda, Maria das Graças Braga Ceccato, Adriano Max Moreira Reis, João Paulo Amaral Haddad, Dirce Inês da Silva, Wânia da Silva Carvalho

**Affiliations:** 1 Universidade Federal de Minas Gerais, Faculdade de Farmácia, Programa de Pós-Graduação Medicamentos e Assistência Farmacêutica, Departamento de Farmácia Social, Belo Horizonte, MG, Brasil.; 2 Universidade Federal de Minas Gerais, Faculdade de Medicina, Belo Horizonte, MG, Brasil.; 3 Universidade Federal de Minas Gerais, Escola de Veterinária, Belo Horizonte, MG, Brasil.; 4 Fundação Hospitalar do Estado de Minas Gerais, Hospital Eduardo de Menezes, Belo Horizonte, MG, Brasil.

**Keywords:** Drug interaction, Antiretroviral therapy, Antitubercular agents, Tuberculosis, HIV

## Abstract

**INTRODUCTION:**

The concomitant use of antituberculosis and antiretroviral drugs, as well as drugs to treat other diseases, can cause drug-drug interactions. This study aimed to describe potential drug-drug interactions (pDDI) in patients with TB and HIV/AIDS co-infection, as well as to analyze possible associated factors.

**METHODS:**

This study was performed in a reference hospital for infectious and contagious diseases in the southeastern region of Brazil and evaluated adult patients co-infected with tuberculosis and HIV/AIDS. A cross-sectional study was conducted in which sociodemographic, clinical, and pharmacotherapeutic characteristics were assessed. The pDDI were identified using the Drug-Reax software. Association analysis was performed using either a chi-squared test or a Fisher’s exact test. Correlation analysis was performed using the Spearman’s coefficient.

**RESULTS:**

The study included 81 patients, of whom 77 (95.1%) were exposed to pDDI. The most frequent interactions were between antituberculosis and antiretroviral drugs, which can cause therapeutic ineffectiveness and major adverse reactions. A positive correlation was established between the number of associated diseases, the number of drugs used, and the number of pDDI. An association was identified between contraindicated and moderate pDDI with excessive polypharmacy and hospitalization.

**CONCLUSIONS:**

We found a high frequency of pDDI, especially among those hospitalized and those with excessive polypharmacy. These findings highlight the importance of pharmacists in the pharmacotherapeutic monitoring in these patients.

## INTRODUCTION

Brazil is among the 20 countries with the highest tuberculosis (TB) and Human Immunodeficiency Virus (HIV) rates in the world^1^. Coinfected patients who are undergoing treatment have a greater potential to develop drug-drug interactions (DDI) that can lead to unfavorable clinical outcomes^2^.

According to the Brazilian guidelines for TB, treatments of new TB cases are based on the combination of four drugs, namely rifampicin (RMP), isoniazid (INH), pyrazinamide (PZA), and ethambutol (ETH), for two months during the intensive phase, and RMP and INH for four months during the maintenance phase^3^. RMP is an enzymatic inducer of cytochrome P450 and thus has the potential to induce pharmacokinetic DDI. INH is an enzymatic inhibitor that may interfere with the hepatic metabolism of other drugs^4^. During the study period, the first-line therapy for acquired immunodeficiency syndrome (AIDS) was tenofovir disoproxil fumarate (TDF), lamivudine (3TC), and efavirenz (EFV) or nevirapine (NVP). Second-line therapy could be used in situations in which the use of EFV or NVP was not possible, thus opting for the use of protease inhibitors^5^. The concomitant use of antiretroviral and other drugs can possibly induce DDI, as many of these induce or inhibit different cytochrome P450 isoenzymes and interfere with several membrane transport proteins, thus influencing the drug absorption and distribution processes^6^.

People infected with HIV are at greater risk of polypharmacy than those who are not infected, mainly due to the development of other diseases that require additional drugs^7^ ^,^ ^8^. In the case of coinfection and/or concomitant diseases, the use of several drugs may lead to DDI and result in adverse reactions or subtherapeutic drug concentration, which may cause the treatment to be ineffective, thus contributing to the appearance of viral resistance and increased health care costs^4^ ^,^ ^9^. 

Nevertheless, studies on DDI in coinfection are scarce, despite its severity, frequency, clinical risk, and evidence level in clinical and pharmacokinetic studies^10^. Hence, the evaluation of potential drug-drug interactions (pDDI) in coinfected patients may support the development of protocols that contribute to appropriate and safe treatments. This may decrease the chances of therapeutic failure, multidrug resistance, and adverse drug reactions. The purpose of this study was to determine the frequency of pDDI in coinfected patients, the association with selected characteristics, and its magnitude in clinical and medical care contexts of patients coinfected with TB and HIV/AIDS.

## METHODS

This was a cross-sectional study conducted between September 2015 and December 2016 in a public hospital, which is a reference center for the treatment of TB, AIDS, and other infectious and contagious diseases, located in Belo Horizonte, Brazil. 

The study population included patients diagnosed with TB and HIV/AIDS, whose TB treatment started in or after September 2015, who agreed to participate in the study, and who were 18 years of age or older. These patients were included only after signing written consent forms.

The sample size was determined considering a sample error of 10%, a 95% confidence interval (CI), and a 50% frequency of potential drug interactions in coinfected patients who received medical care at the hospital in 2014. The number of coinfected patients in that year was 136. The calculated sample size was 57 patients. However, considering a 30% refusal rate, after conducting a pilot study, the minimum calculated sample size was 74 patients.

The data were collected through patient interviews, together with a search conducted by the research pharmacist in the patients’ medical and prescription records regarding sociodemographic, clinical, and pharmacotherapeutic characteristics. 

The pDDI were identified using the Drug-Reax® software (Truven Health Analytics, Greenwood Village, Colorado, USA)^11^. An interaction is called pDDI when it corresponds to a DDI that can theoretically occur during the patient’s pharmacotherapy. In the present study, the term pDDI will be employed to refer to potential drug-drug interactions.

The identification of the pDDI was performed after data collection, which made it impossible to recommend interventions to optimize pharmacotherapy and analyze the clinical manifestations of the interactions.

The Drug-Reax software (Truven Health Analytics, Greenwood Village, Colorado, USA)^11^ provides information on the potential clinical consequences or adverse reactions to drugs resulting from the interaction and rates the pDDI with regard to the severity and level of documented evidence. 

The pDDI are rated according to four categories of severity:

- Contraindicated: concurrent use of the drugs is contraindicated.
- Major: the interaction may be life-threatening and/or require medical intervention to minimize or prevent serious adverse events.
- Moderate: the interaction may result in exacerbation of the patient's condition and/or require an alteration in therapy.
- Minor: This interaction has limited clinical effects. The manifestations may include an increase in the frequency or severity of the side effects; however, in general, they do not require a major alteration in therapy.

Regarding the level of documented evidence, the DDIs are rated as:

- Excellent: the interactions have been proven by controlled studies.
- Good: The documentation vehemently suggests that the interaction exists, but controlled studies performed in an appropriate manner are insufficient.
- Fair: either the available documentation is unsatisfactory, but pharmacological considerations lead physicians to suspect the existence of interaction, or the documentation is good for a pharmacologically similar drug.

The main dependent variable was the occurrence of pDDI, regardless of the severity. The other dependent variables were the occurrence of pDDI considering the severity: contraindicated, major, and moderate.

The independent variables were divided into sociodemographic, clinical, and pharmacological categories. Sociodemographic variables included gender and age (≤40 or >40 years, stratified by the median). Clinical variables included the TB clinical form (pulmonary, extrapulmonary, pulmonary + extrapulmonary), TB treatment time up to two months (yes or no), HIV diagnosis time up to one year (yes or no), associated diseases (yes or no), detectable viral load (yes or no), CD4+ >200 cells/mm^3^ (yes or no), and hospitalization (yes or no). Pharmacological variables included the number of drugs used, TB therapy, antiretroviral therapy (yes or no), use of rifampicin (yes or no), and excessive polypharmacy (no, if <10 drugs or yes if ≥10 drugs)^12^. 

The data were recorded in Excel 2007 worksheets. Descriptive analysis was conducted by applying the categorical variable frequency distribution and by employing central tendency (mean and median) and dispersion (standard deviation and interquartile range) measures for quantitative variables. 

The association between dependent and independent categorical variables was assessed using either the chi-squared test or Fisher's exact test, if appropriate. 

The correlation between the number of drugs, the number of associated diseases, and the number of pDDI was determined by applying Spearman's non-parametric test. 

For all analyses performed in this study, statistical significance was set at p<0.05. Statistical analyses were performed using the Windows Statistical Package for the Social Sciences (SPSS), version 21.0. 

### Ethical Consideration

This study was approved by the Research Ethics Committee of Universidade Federal de Minas Gerais (UFMG) (CAAE: 23692713.3.0000.5149) and the Minas Gerais State Hospital Foundation (FHEMIG) (CAAE:23692713.2.3001.5124).

## RESULTS

**FIGURE 1:**
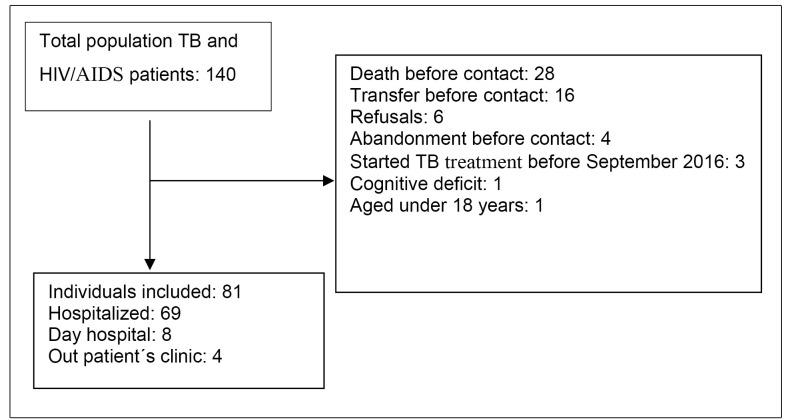
Flowchart outlining the number of patients coinfected with tuberculosis and HIV/AIDS included in the study.

Of the 140 patients with TB and HIV/AIDS coinfection between September 2015 and December 2016, 59 were excluded for different reasons (Figure 1). There were six refusals.

This study included 81 patients with TB and HIV/AIDS coinfection, of which 77% were men, with a median age of 40 years (interquartile range, IQR: 33-48). The median number of drugs was 13 (IQR: 11.5-17.5), and 85% of the patients exhibited excessive polypharmacy. This study found that the most frequently used TB therapy was RMP, INH, PZA, and ETH, and 88% of the patients were in the intensive treatment phase. The first-line treatment at the time of the study was administered to 38% of the patients. The other patient characteristics are listed in Table 1. 

**TABLE 1:** Descriptive review of sociodemographic, clinical, and pharmacotherapeutic characteristics of included study participants (n=81).

Characteristics	Total of patients	
	n	%
**Sociodemographic**		
Men	62	77
≤ 40 years old	42	52
**Clinical form of tuberculosis**		
Pulmonary	46	57
Extrapulmonary	27	33
Pulmonary + extrapulmonary	8	10
**TB treatment time**		
Up to two months	71	88
**HIV diagnosis time**		
Up to one year	44	54
**Associated diseases**		
None	18	22
Candidiasis	19	23
Pneumonia	10	12
Cytomegalovirus infection	8	10
Thyroid disorders	6	7
Hepatitis	6	7
Syphilis	6	7
**Viral Load**		
Detectable	71	88
**CD4 T lymphocyte count**		
<200	59	73
**TB therapy**		
Basic (RMP, INH, PZA, ETH)	69	85
Special (Rifabutin, SM, OFX, ETH, or Levofloxacin)	12	15
**Antiretroviral therapy**		
None	40	49
TDF+3TC+EFV or nevirapine	31	38
TDF+3TC+ IP/r	4	5
Others	6	7
**Number of drugs**		
5-9	12	15
≥10	69	85

**TB:** tuberculosis; **RMP:** rifampicin; **INH:** isoniazid; **PZA:** pyrazinamide; **ETH:** ethambutol; **SM:** streptomycin; **OFX:** ofloxacin; **TDF:** tenofovir; **3TC:** lamivudine; **EFV:** efavirenz.

In this study, 95.1% of patients exhibited pDDI. Considering their severity rating, major pDDI occurred in 82.7% of the patients, moderate pDDI in 72.8%, minor pDDI in 54.3%, and contraindicated pDDI in 24.7%.

The median number of pDDI per patient was 3 (IQR: 2-7.5). A positive correlation was found between the number of drugs used and pDDI (Spearman's rho= 0.703, p <0.0005). A positive correlation was also detected between pDDI and the associated diseases (Spearman's rho= 0.55, p <0.0005).

Patients who exhibited contraindicated pDDI proved to be inpatients under excessive polypharmacy use. Antiretroviral therapy was associated with major pDDI. Most patients with moderate pDDI were inpatients under excessive polypharmacy and presented with associated diseases (Table 2).

**TABLE 2:** Analysis of the selected characteristics' association with the presence of contraindicated, major, and moderate interactions (n=81).

		All interactions		Contraindicated		Major		Moderate	
Characteristics		Yes %	No %	Yes %	No %	Yes %	No %	Yes %	No %
Age	≤40	38 47	4 5	8 10 ^a^	34 42	34 42 ^a^	8 10	30 37 ^a^	12 15
	>40	39 48	0 0	12 15	27 33	33 41	6 7	29 36	10 12
									
Excessive polypharmacy	Yes	67 83	2 2	20 25*	49 60	58 72	11 14	57 70*	12 15
	No	10 12	2 2	0 0	12 15	9 11	3 4	2 2	10 12
									
Use of ART	Yes	40 49	1 1	13 16 ^a^	28 35	38 47* ^a^	3 4	29 36^a^	12 15
	No	37 46	3 4	7 9	33 41	29 36	11 14	30 37	10 12
									
Use of Rifampicin	Yes	66 81	4 5	18 22	51 63	57 70	12 15	51 63	18 22
	No	11 14	0 0	2 2	10 12	10 12	2 2	8 10	4 5
									
Hospitalization	Yes	68 84	1 1	20 25*	49 60	58 72	11 14	56 69*	13 16
	No	9 11	3 4	0 0	12 15	9 11	3 4	3 4	9 11
									
Associated diseases	Yes	62 77*	1 1	17 21	46 57	53 65	10 12	53 65*	10 12
	No	15 19	3 4	3 4	15 19	14 17	4 5	6 7	12 15

* p<0.05, presence of interaction compared to absence of interaction. ^a^ Comparisons were made using the chi-squared test.

Other comparisons were made using the Fisher's exact test. **ART:** antiretroviral therapy.

In the present study, 423 pDDI cases were identified. Antituberculosis drugs were involved in 49% of patients, and antituberculosis and antiretroviral drugs were present in 12%, while 37% involved antituberculosis drugs and drugs for the treatment of other diseases.

Regarding severity, 6.6% of the pDDI were contraindicated, 12.1% were minor, 38.5% were major, and 42.8% were moderate. Considering the level of documentation, 14.7% were excellent, 34.5% were good, and 50.8% were fair.

Table 3 provides the pDDI characteristics of antituberculosis and antiretroviral drugs in relation to severity, frequency, clinical risk, and level of documentation. The most frequent pDDI were EFV and RMP, and RMP with ritonavir.

**TABLE 3:** Description of the most frequent drug interactions involving antituberculosis and antiretroviral drugs.

Interactions	Severity	n	%	Risk	Level of documentation
Rifampicin +Antiretroviral drugs					
Rifampicin +Efavirenz	Major	33	41	Decreased EFV effectiveness	Regular
Rifampicin+ritonavir	Contraindicated	3	4	Decreased ritonavir concentration	Good
Rifampicin +Atazanavir	Contraindicated	2	2	Decreased atazanavir concentration	Excellent
Rifampicin+Nevirapine	Major	1	1	Decreased nevirapine concentration	Excellent
Rifampicin+saquinavir	Contraindicated	1	1	Decreased saquinavir effectiveness and increased hepatotoxicity	Excellent
Rifampicin+zidovudine	Moderate	1	1	Decreased zidovudine concentration	Good
Rifabutin + Antiretroviral drugs					
Rifabutin +ritonavir	Major	2	2	Increased rifabutin concentration	Excellent
Rifabutin+atazanavir	Major	1	1	Increased rifabutin concentration	Good
Rifabutin+fosamprenavir	Major	1	1	Increased rifabutin concentration	Excellent
Quinolones + Antiretroviral drugs					
Levofloxacin +ritonavir	Major	1	1	Increased risk of QT interval alteration	Regular
Ofloxacin+Efavirenz	Major	2	2	Increased risk of QT interval alteration	Regular

**EFV:** Efavirenz.

The most frequent major pDDI included EFV and RMP, fluconazole, sulfamethoxazole, and trimethoprim, and INH and paracetamol, while prednisone and RMP, fluconazole and RMP, as well as omeprazole and RMP (Table 4).

**TABLE 4:** Description of the most frequent major and moderate drug interaction characteristics.

Interactions	Severity	n %	Risk	Level of documentation
**Severe**				
Efavirenz+Rifampicin	Major	33 41	Decreased EFV effectiveness	Excellent
Fluconazole+Sulfamethoxazole	Major	13 16	Increased risk of QT interval alteration	Regular
Isoniazid+Paracetamol	Major	10 12	Increased hepatotoxicity risk	Excellent
Azithromycin+Efavirenz	Major	7 9	Increased risk of QT interval alteration	Regular
Moderate				
Prednisone +Rifampicin	Moderate	21 26	Decreased prednisone effectiveness	Good
Fluconazole+Rifampicin	Moderate	16 20	Decreased fluconazole effectiveness	Excellent
omeprazole+Rifampicin	Moderate	15 19	Decreased omeprazole effectiveness	Regular
Fluconazole +Prednisone	Moderate	9 11	Increased prednisone effectiveness	Good
Diazepam+Rifampicin	Moderate	8 10	Decreased diazepam effectiveness	Good
Diazepam +Isoniazid	Moderate	7 9	Increased risk of diazepam toxicity	Good

**EFV:** Efavirenz.

Table 5 provides the characteristics of contraindicated pDDI in terms of their frequency, clinical risk, and level of documentation. The most frequent pDDI were amitriptyline and metoclopramide, clarithromycin, and sulfamethoxazole, as well as haloperidol and metoclopramide.

**TABLE 5:** Description of the most frequent contraindicated interactions (n=81).

Interactions	Severity	n %	Risk	Level of documentation
Amitriptyline+metoclopramide	Contraindicated	5 6	Extrapyramidal reactions and neuroleptic malignant syndrome	Regular
Clarithromycin+sulfamethoxazole	Contraindicated	4 5	Increased risk of QT interval alteration	Good
Haloperidol+metoclopramide	Contraindicated	4 5	Extrapyramidal reactions and neuroleptic malignant syndrome	Regular
Clarithromycin+fluconazole	Contraindicated	3 4	Increased risk of QT interval alteration	Good
Fluconazole+haloperidol	Contraindicated	3 4	Increased risk of QT interval alteration	Regular
Rifampicin+ritonavir	Contraindicated	3 4	Decreased ritonavir concentration	Good
Atazanavir+rifampicin	Contraindicated	2 2	Decreased atazanavir concentration	Excellent
Metoclopramide+risperidone	Contraindicated	2 2	Extrapyramidal reactions and neuroleptic malignant syndrome	Regular
Carbamazepine+Efavirenz	Contraindicated	1 1	Reduced EFV plasmatic concentration	Excellent
Dapsone+Saquinavir	Contraindicated	1 1	Increased risk of QT interval alteration	Regular
Fluconazole+Ondansetron	Contraindicated	1 1	Increased risk of QT interval alteration	Regular
Fluconazole+ritonavir	Contraindicated	1 1	Increased risk of QT interval alteration	Regular
Fluoxetine +metoclopramide	Contraindicated	1 1	Extrapyramidal reactions and neuroleptic malignant syndrome	Excellent
Rifampicin+saquinavir	Contraindicated	1 1	Decreased saquinavir effectiveness and increased hepatotoxicity	Excellent

## DISCUSSION

This is one of the first studies to investigate pDDI in TB and HIV/AIDS-coinfected patients, considering all drugs used by patients. Some studies have evaluated pDDI with antiretroviral drugs^13^ ^,^ ^14^ ^,^ ^15^ ^,^ ^16^ ^,^ ^17^ ^,^ ^18^ ^,^ ^19^ ^,^ ^20^ ^,^ ^21^ ^,^ ^22^, and a small number identified pDDI with anti-TB drugs^23^.

It was observed that pDDI in antiretroviral therapy is common, varying from 23 to 41% in different studies^15^ ^,^ ^18^ ^,^ ^19^. In patients with TB, pDDI mainly involve RMP^23^. In the present study, the global prevalence of pDDI was much higher than that considered in studies involving monoinfected patients. Moreover, one should consider the magnitude of the interaction in the clinical context of medical care provided to patients with infectious diseases, in terms of severity and potential associated adverse events to subsidize the clinical follow-up of pDDI.

The population of this study is characterized as being seriously ill, newly diagnosed with HIV, and most with CD4 count below 200 cells/mm^3^. This may be associated with a late diagnosis of HIV and may interfere with the number of diseases presented by patients, considering that the more severe the immunosuppression, the greater the chance of occurrence of opportunistic diseases. This agrees with our results in that moderate and contraindicated pDDI were associated with excessive polypharmacy and hospitalization. Prophylaxis is necessary because it can prevent the development of some diseases and, subsequently, curb the increase in the number of medications, hospitalizations, and pDDI^7^.

Polypharmacy represents a challenge in the management of pharmacotherapy, especially among patients with multiple diseases^7^. In accordance with a prior study of DDI in patients with HIV^24^, a positive association was identified between the number of drugs, the number of associated diseases, and the number of pDDI.

The most frequent pDDI was between RMP and EFV, which is an important interaction, as it can reduce EFV concentration by 20%-25%. This is because RMP is an inducer of CYP2B6 and CYP3A4, which are involved in drug metabolism, leading to therapeutic failure and the selection of drug-resistant viral strains^10^. At the time of this study, antiretroviral regimens composed of two nucleoside reverse transcriptase inhibitors (NRTI) + EFV constituted the principal option of ART for patients using RMP^5^. The current recommendation to bypass the association between drugs for tuberculosis and HIV/AIDS is the use of integrase inhibitors^25^. These drugs provide a genetic barrier and facilitate faster viral suppression. However, pDDI in this class are also important for evaluation, as they are drugs that interact with RMP and need to be used twice a day, which can compromise adherence to ART^26^. The concomitant use of these drugs could not be avoided, but these patients need to be followed more carefully to detect viral escape, hepatotoxicity, and neurotoxicity. 

A clinical manifestation of the interaction between RMP and EFV is the increase in the 8-hydroxy efavirenz metabolite, which is associated with neurotoxicity, especially in women and individuals with the CYP2B6* 6 polymorphism^27^. The neurotoxic effect of prolonged exposure to 8-hydroxy efavirenz during the combined treatment of EFV with RMP needs to be better explained^28^. However, in clinical practice, this potential adverse effect should be considered until adverse drug reactions can be further studied.

The clinical relevance of interactions between RMP and protease inhibitors has a negative impact on the effectiveness of antiretroviral treatment, as it reduces the plasma concentration of the antiretroviral drug by up to 75%^4^. Although this association is contraindicated and well documented in the Brazilian protocols for coinfection treatment^3^, as well as in publications about DDI in HIV patients^4^, the current study was able to detect the prescription of protease inhibitors with RMP. The use of alternative therapy without RMP may have unfavorable outcomes, as this is the most powerful antituberculosis drug^29^. Therefore, patients exhibiting this interaction should be assessed for the feasibility of using rifabutin or an antiretroviral drug of a different therapeutic class, as integrase inhibitors^25^. Currently, the first alternative for the treatment of HIV/AIDS in patients with TB is the use of dolutegravir or raltegravir in cases of contraindication to dolutegravir^25^ ^,^ ^26^.

The reduced therapeutic effectiveness due to the inclusion of RMP in pharmacotherapy also occurs with fluconazole, which is used to treat candidiasis, a common opportunistic infection in HIV patients. However, other antifungal drugs may be used as appropriate therapeutic alternatives, thus illustrating the importance of identifying clinically relevant DDIs in the pharmacotherapy of coinfected patients. A large proportion of the patients presented with a low lymphocyte T CD4 count, which indicates severe immunodeficiency^5^, thus reinforcing the importance of evaluating the effectiveness of pharmacotherapy.

Hepatotoxicity, an adverse drug reaction that may occur in patients using antituberculosis drugs, may be enhanced by DDI with antiretroviral drugs (saquinavir and RMP) or with drugs used to treat symptoms or associated diseases (paracetamol and RMP). In this case, pharmacotherapy safety assessment through the monitoring of hepatic enzymes should be encouraged to determine the most appropriate conduct^3^. 

Another major adverse drug reaction that may be induced by DDIs is an increased QT interval. Drugs that prolong the QT interval are important in clinical practice because of the risk of cardiotoxicity with *torsades de pointes* and cardiac arrest^30^. These adverse drug reactions may be determined by pharmacokinetic pDDI, which inhibits the metabolism of drugs with this property, or by pharmacodynamic synergism. The pDDI of EFV and ofloxacin, levofloxacin and ritonavir, fluconazole and sulfamethoxazole, azithromycin and EFV, clarithromycin and sulfamethoxazole, clarithromycin, and fluconazole, fluconazole, and haloperidol, dapsone and saquinavir, fluconazole and ondansetron, as well as fluconazole and ritonavir in this investigation may cause the adverse drug reactions mentioned above. Therefore, in treating coinfected patients, it is important to know the drugs that prolong the QT interval, as well as the other risk factors that contribute to this reaction, to adopt the most appropriate strategies to handle and monitor pDDI effects.

The benefits of a pharmacist’s action in improving the clinical results for HIV patients have been described in different studies^31^ ^,^ ^32^ ^,^ ^33^ ^,^ ^34^. A systematic review assessed the impact of clinical pharmacists in HIV patients to demonstrate progress and to understand the expertise required to minimize DDI, contraindications, and adverse reactions. Given the growing complexity of HIV treatment, pharmacists trained in HIV pharmacotherapy are invaluable to the multidisciplinary care team^35^. Therefore, pharmacists play an important role in patient education, monitoring effectiveness, pharmacotherapy safety, and promoting rational drug use^36^.

The limitations of this study included the absence of analysis of the clinical consequences of DDI, as the data analysis was performed after the data had been collected, having used only one source for analysis, and the fact that the study was conducted in only one reference center for the treatment of infectious and contagious diseases. One strength of this study was that it used a pDDI software that presents an appropriate sensitivity and specificity for use in pharmaco-epidemiological studies as well as in clinical practice^37^. However, the identification of drug interactions via software generally produces a high signal level, which can identify a greater frequency of pDDI^38^.

Although this study has limitations, understanding the pDDI and its magnitude applied to these patients is crucial for the proper monitoring of pharmacotherapy.

The frequency of pDDI in coinfected patients has increased, especially among drugs used to treat TB and HIV/AIDS. An association was identified between contraindicated and moderate pDDI with excessive polypharmacy and hospitalization. Drug interactions have the potential to induce therapeutic failures and severe adverse reactions, such as neurotoxicity, hepatotoxicity, and increased QT intervals. These findings highlight the importance of pharmacists in pharmacotherapy monitoring in these patients.
